# Investigation of the relationship between early maladaptive schemas, temperament and eating attitude in adults

**DOI:** 10.1186/s40337-022-00711-w

**Published:** 2022-11-29

**Authors:** Kahraman Güler, Zeynep Özgörüş

**Affiliations:** Psychology Department, Faculty of Economics and Administrative Social Sciences, Istanbul Gelisim University, Istanbul, Turkey

**Keywords:** Early maladaptive schemas, Temperament, Eating attitude

## Abstract

**Background:**

Current research on eating attitude has focused primarily on female perspective (Kapoor et al. in J Educ Health Promot 11(80):1–7, 2022; Piko et al. in J Prev Med Hyg. 63(1):83–89, 2022). To extend cross-gender approaches, this study aimed to examine the relationship between early maladaptive schemas, temperament, and eating attitude and to see whether these three concepts differ between men and women.

**Methods:**

The sample group consists of a total of 308 participants, 206 (66.9%) women and 102 (33.1%) men, living in Bursa and participating in the research voluntarily. In the study, Sociodemographic Data Form was used to obtain personal information of the participants, Young Schema Questionnaire—Short Form 3 was used to evaluate early maladaptive schemas, Temperament Evaluation of Memphis, Pisa, Paris and San-Diego Autoquestionnaire was used to evaluate temperament characteristics and Eating Attitude Test was used to evaluate eating attitudes.

**Results:**

As a result of the study, a moderate and positive relationship was found between the Defectiveness, Emotional Inhibition, Enmeshment/Dependence, Failure, Abandonment, Vulnerability to Harm or Illness, Negativity/Pessimism schemas and eating attitude. Among these schemas, Defectiveness was found to be the best predictor of deterioration in eating attitude. A weak and positive relationship was found between the scores obtained from the Eating Attitude Test and Cyclothymic and Irritable temperaments, and a moderate positive relationship with Anxious temperament. Among these temperaments, Anxious temperament was found to be the best predictor of deterioration in eating attitude.

**Conclusions:**

When the results were examined, a relationship was observed between early maladaptive schemas and temperament types, and this relationship was examined in detail. The results obtained at the end of the study were discussed and suggestions were made for future studies.

## Plain English Summary

This study examined the relationship between early maladaptive schemas, temperament, eating attitude and the effect of gender variable on these concepts in adults. Early maladaptive schemas are basic mental constructs that occur as a result of negative experiences early in life. Temperament is a long-term, often life-long structural traits that change little throughout an individual’s life. Eating attitude is the tendency that creates an individual’s feelings, thoughts and behaviors related to eating and nutrition. Disruptions in eating attitudes can lead to eating disorders. Results revealed a relationship between Defectiveness, Emotional Inhibition, Enmeshment/Dependence, Failure, Abandonment, Vulnerability to Harm or Illness, Negativity/Pessimism schemas and eating attitude. Among schemas, Defectiveness was found to be the best predictor of deterioration in eating attitude. Among temperaments, Anxious temperament was found to be the best predictor of deterioration in eating attitude. Additionally, a relationship was found between early maladaptive schemas and temperament types. This relationship sheds light on the relationship between the development and treatment of eating disorders.

## Introduction

Individuals begin to organize their experiences with an effort to make sense of the world. They try to establish an order within these complex experiences, objects and stimuli. The regulatory framework and patterns that help create this order are called "schemas". Schemas are formed early in life and continue to take shape throughout life [3]. Three main considerations are factors in the maladaptive formation that occurs as a result of the damage to the needs in childhood. These are the thoughts about the future, the world and the self [4 as cited in 5].

Universally, every human being has needs. Giving individuals too much of these needs is just as damaging [3]. Rafaeli et al. [3] summarized these needs as follows: stability, autonomy, acceptance, safety, competition, sense of self, freedom to express one's feelings and needs, realistic boundaries, spontaneousness, and play. When schemas that enable us to cope with situations are misinterpreted, they lose their functionality and can be disorienting, maladaptive schemas affect the progression of experiences and can cause the individual to lose flexibility as they become more rigid over time.

Four main experiences in childhood are an important factor in the formation of early maladaptive schemas. The four main experiences mentioned are as follows: The child's detrimental inhibition of needs, selective internalization and identification with significant others, traumas that make the child feel and/or experience the presence of danger and threat in a painful way, and finally, excessive giving of good things [3]. Young et al. [6] defined rigid beliefs and patterns consisting of bodily senses, memories, emotions and cognitions that develop in childhood or adolescence, take into account the individual's relationships with herself or others, as "Early Maladaptive Schemas".

Young et al. [6] identified 18 schemas: Abandonment/Instability, Mistrust/Abuse, Emotional Deprivation, Defectiveness/Shame, Social Isolation/Alienation, Dependence/Incompetence, Vulnerability to Harm or Illness, Enmeshment/Undeveloped Self, Failure, Entitlement/Grandiosity, Insufficient Self-Control/Self-Discipline, Subjugation, Self-Sacrifice, Approval-Seeking/Recognition-Seeking, Negativity/Pessimism, Emotional Inhibition, Unrelenting Standards/Hypercriticalness, and Punitiveness and 5 domains: Disconnection and Rejection, Impaired Autonomy and Performance, Impaired Limits, Other-Directedness, and Overvigilance and Inhibition. Schemas in Disconnection and Rejection domain occur when the person experiences disconnections and long separations in the family environment, has unstable, rejecting, abusive, unaffectionate and easily angered parents [3] and the child's attachment needs are disappointed [7]. Schemas in Impaired Autonomy and Performance domain are found in individuals who are given too much attention by the caregiver early in life and who do not have space to develop their skills, or who are not cared for or given too little attention. Individuals have difficulty in forming their own identities, establishing their own lives and being self-sufficient. Schemas in Impaired Limits domain originate from the behaviors that are too liberal and tolerant [6]. Schemas in Other-Directedness domain are formed in childhood when individual grow up in an environment of conditional love, respect and acceptance. Lastly, schemas in Overvigilance and Inhibition domain arise due to the harsh approach of caregivers [3].

A person’s activity level, compatibility, modes and thoughts having a variable structure and long-term regular biological essence of the personality are defined as “temperament” [8]. In summary, temperament refers to long-term, generally life-long tendencies that symbolize individual. Temperament may be a harbinger of a mental disorder or may be excessive in a way that can be considered statistically abnormal even in the absence of a mental disorder [9].

Kreapelin, who was the first person to deal with the concept of temperament in clinical psychiatry, defined 4 basic emotions as depressive, cyclothymic, hyperthymic and irritable temperament. Kreapelin suggested a relationship between affective temperament and affective pathology [10]. Akiskal and Mallya added the anxious temperament type to Kreapelin's theorem and created the "Affective Temperament Model" [11]. They defined five temperament categories in the affective temperament model. These are: depressive temperament, irritable temperament, hyperthymic temperament, cyclothymic temperament and anxious temperament [11].

Individuals with cyclothymic temperament are characterized by sudden shifts from one stage to the next, each stage having infrequent euthymia that lasts for days. Decreased sleep versus oversleeping; introversion versus extroversion; talkativeness versus silence, excessive playfulness versus inexplicable crying, drowsiness, and somatic discomfort versus hyperactivity; sharp perceptions against atrophy of the senses; sharp perceptions against slowing understanding, self-esteem oscillating between low self-esteem versus overconfidence; anxiety from pessimism versus anxiety from optimism [12].

Individuals with hyperthymic temperament are generally adaptable, over-optimistic, cheerful, extroverted, over-talkative, over-confident, playful, high-energy, irritable, seeking novelty and stimuli. In addition to this, intermittent sub-threshold hypomanic symptoms are seen with intermittent euthymia in the temperament characteristics of individuals. It is common for these individuals to sleep less than 6 h a day and to use denial defense mechanisms [13].

Individuals with a depressive temperament usually have anxious and pessimistic thoughts. These people sleep more than 9 h a day. They tend to have anhedonia and less psychomotor energy in the morning hours with this they are introverted, incapable and unsuccessful, overly anxious, complaining, often having critical attitudes towards themselves and blaming, and low libido. These individuals are also calm, reliable and self-disciplined characters [9].

Individuals with an irritable temperament are prone to brooding, irritable and rarely euthymic, often characterized by pessimism. These people often have features such as restlessness, expressiveness of intense emotions, sarcasm, dominant, dysphoria, impulsivity and a critical attitude towards others [14].

Individuals with an anxious temperament have worries about daily routines, feelings of insecurity, and premonitions about misfortune. They are usually in a state of alertness and have difficulty in relaxing. Anxious temperament is divided into 3 types as anxious-avoidant, anxious-phobic, anxious-sensitive temperament [9].

Nourishment is a very important need for human existence. “Eating” is an innate human impulse and an indispensable need [15]. Eating, on the other hand, is one of the behaviors that should be experienced by people to continue their lives and that gives people pleasure. Eating begins with birth and is a behavior that interacts with different variables throughout life. Attitudes are part of an individual's feelings, thoughts and psychological behaviors [16] and are the tendency of the individual to constantly react to the situations around him in a similar way [17]. Therefore, eating attitudes are the tendencies that are effective in the formation of similar thoughts, behaviors and feelings that a person shows towards nutrition and eating [16]. Disruption of this tendency towards nutrition and eating can lead to deterioration of eating attitudes. Familial factors, biological-psychological predisposition, interaction of negative social conditions [18], childhood traumas [19], body image, genetic and biological factors [20] are accepted as risk factors in the development of these deteriorated eating attitudes.

The increase in eating disorders in recent years has led to an increase in research and discussions on eating attitudes. However, studies have shown that changes and deterioration in eating behavior can have many causes. Various studies have shown that temperament and early maladaptive schemas may contribute to deterioration in eating attitudes. It is thought that affective temperament also plays an important role in the development and maintenance of eating disorders.

This study aims to examine eating attitudes in the light of Akiskal's affective temperament model and Young's concepts of early maladaptive schemas and to understand the relationship between these three concepts. Some of the studies show that the reason for the deterioration in eating behavior and attitude of people can be developed as a coping strategy with early maladaptive schemas [21]. Therefore, examining the relationship between increasing eating disorders and early maladaptive schemas will contribute to the literature and enable more effective use of schemas in the treatment of eating disorders. In addition, it is thought that determining the mediating factors of the relationship between early maladaptive schemas, temperament and eating attitudes will provide prevention, understanding of the causes of eating disorders and early intervention.

## Methods

### Design

In this study, the relational screening model was used to examine the relationship between early maladaptive schemas, eating attitudes and temperament, and the relationship between the sociodemographic characteristics of individuals and the status of the variables.

### Participants

A random sampling method based on households in Bursa was used to select participants. From the selected households, one adult over the age of 18 was randomly selected for this study resulting in 308 participants (M = 30). Participants were able to reject participating in this study as participation was voluntary. Among these participants, 33.1% are male and 66.9% are female. 54.5% participants are working and 45.5% are not working. 6.8% participants are primary school graduates, 8.1% are secondary school graduates, 24% are high school graduates, 51.9% are university graduates and 9.1% are graduates (specialist status) and above. 71.4% participants are diagnosed with a mental illness by a psychiatrist and 28.6% are not. Participants who are diagnosed with a mental illness did not want to share what type of mental illness they have. Participants’ names and surnames were not taken to increase the validity and reliability of the data collection tools.

### Procedure

Participants completed self-administered anonymous questionnaires (Personal Information Form, TEMPS-A Temperament Scale, Eating Attitude Test and Young Schema Questionnaire—Short Form 3) in the presence of a research assistant. Ethical approval was obtained from the Ethics Committee of Aydin University.

### Measures

In the study, Personal Information Form to obtain personal information of the participants, Eating Attitude Test to detect if there is deterioration in eating attitudes, Young Schema Questionnaire Short Form-3 (YSQ-SF3) to evaluate early maladaptive schemas and determine the scores obtained from the schemas, TEMPS-A Temperament Scale to evaluate affective temperament characteristics.

#### Personal Information Form

Participants were given a Personal Information Form prepared by the researchers, and questions asked about their gender, age, mental illness and educational status.

#### The Eating Attitude Test

The Eating Attitude Test, developed by Garner and Garfinkel [22] to examine abnormalities in eating attitudes, is a self-report scale [23]. The adaptation and standardization of the scale into Turkish was done by Savaşır and Erol [24] and the cut-off point was determined as 30 [25]. Test–retest reliability was stated as 0.65 and Cronbach Alpha internal consistency coefficient as 0.70. In Batur's study [26], the factor structure of the scale items was processed separately for women and men, and the Cronbach alpha internal consistency coefficients were evaluated. The stated 4 factor values were between 0.47 and 0.90 in women; It was found that the factor structures in men varied between 0.34 and 0.80. The Cronbach Alpha Coefficient, which is the internal consistency value for this study, was found to be 0.83 for The Eating Attitude Test.

#### Young Schema Questionnaire—Short Form 3 (YSQ-SF3)

In the study, version 3 of the short form of the Young Schema Questionnaire was used to identify early maladaptive schemas in adults. The aim of the scale is to evaluate the schemas that occur as a result of not meeting the general needs of childhood properly. Young Schema Questionnaire—Short Form 3 is the most recently developed short form of 90 items, which includes 18 schemas as a result of the development of the Young Schema Questionnaire [27]. The Turkish adaptation of the Young Schema Questionnaire Short Form 3 was performed on a university population in 2009 by Soygüt et al. [27]. The conclusion reached as a result of the analyzes made is that the validity and reliability of the developed scale is at a sufficient level. As a result of the basic cognitive analyzes for construct validity, it was seen that the 14-factor construct was more valid than the 5 schema domains. This 14-factor includes emotional deprivation, social isolation/mistrust, defectiveness/shame, emotional inhibition, enmeshment/dependence, abandonment, vulnerability to harm or illness, failure, negativity/pessimism, entitlement/insufficient self-control, self-sacrifice, punitiveness, unrelenting standards and approval-seeking. It was stated that the internal consistency coefficient of the scale was 0.95 and the internal consistency coefficients of the subscales were between 0.54 and 0.85 [28]. The Cronbach Alpha Coefficient, which is the internal consistency value for this study, was found to be 0.73 for approval-seeking, 0.67 for unrelenting standards, 0.64 for punitiveness, 0.70 for self- sacrifice, 0.76 for entitlement/insufficient self-control, 0.80 for negativity/pessimism, 0.74 for failure, 0.73 for vulnerability to harm or illness, 0.71 for abandonment, 0.77 for enmeshment/dependence, 0.74 for emotional inhibition, 0.71 for defectiveness/shame, 0.81 for social isolation/mistrust, 0.75 for emotional deprivation.

#### Temperament Evaluation of Memphis, Pisa, Paris, San Diego Autoquestionaire (TEMPS-A)

The TEMPS-A Temperament Scale was designed by Akiskal et al. [13]. To evaluate the dominant affective temperament. The original version of the scale consisted of 110 items for women and 109 items for men. There are 99 items in the Turkish version. The Turkish validity and reliability study of the scale was performed by Vahip et al. [29]. Test–retest reliability is between 0.73 and 0.91, Cronbach Alpha internal consistency coefficient is between 0.75 and 0.84 [30]. It consists of 5 sub-dimensions that determine anxious, depressive, irritable, cyclothymic and hyperthymic temperaments [31]. The Cronbach Alpha Coefficient, which is the internal consistency value for this study, was found to be 0.82 for depressive temperament, 0.88 for cyclothymic temperament, 0.84 for hyperthymic temperament, 0.90 for irritable temperament, 0.87 for anxious temperament.

## Statistical methods

After the data were transferred to the SPSS 25 program, the analyzes were started. According to Groeneveld and Meeden [32], Moors [33], Hopkins and Weeks [34] and De Carlo [35], coefficients of kurtosis and skewness values between − 3 and + 3 provide a normal distribution and at the same time according to George and Mallery [36], skewness and kurtosis values will be sufficient between − 2 and + 2. Relevant kurtosis and skewness values are shown in Table 1. After deciding on the normal distribution, Independent Samples T-Tests were conducted to compare men versus women on all variables of interest (Eating Attitude Test, TEMPS-A, Young Schema Questionnaire). Multiple Linear Regression analysis was performed to analyze the predictive variable of the independent variable(s) and it was preferred to establish the model using the Stepwise method. I—Variables are quantitative (at least on the interval scale) and show normal distribution. II—There should be a linear relationship between dependent and independent variables. III—The Variance Inflation Factor (VIF) of a linear regression gives us an idea of how much the variance of the regression estimates is due to the medium linearity of its accuracy. Variance Magnification (VIF) of each independent is less than 10; Tolerances must be greater than 0.2 (or 0.1 in some sources). IV—As a result of the regression analysis, the differences/residues (estimate errors) between the predicted values and the observed values should exhibit a normal distribution. After controlling these assumptions, Multiple Linear Regression analysis was performed.

**Table 1:** Skewness and kurtosis values for Eating Attitude Test, TEMPS-A, Young Schema Questionnaire

	n	Skewness	Kurtosis
Eating Attitude Test	308	1.536	2.248
TEMPS-A			
Depressive Temperament	308	0.793	0.125
Cyclothymic Temperament	308	0.113	− 1.097
Hyperthymic Temperament	308	− 0.204	− 0.919
Irritable Temperament	308	0.959	0.044
Anxious Temperament	308	0.600	− 0.626
Young Schema Questionnaire			
Emotional Deprivation	308	1.473	1.931
Social Isolation/Mistrust	308	0.975	0.923
Defectiveness/Shame	308	1.442	1.647
Emotional Inhibition	308	0.791	0.277
Enmeshment/Dependence	308	1.165	1.056
Abandonment	308	1.455	1.829
Vulnerability to Harm or Illness	308	0.893	0.623
Failure	308	1.430	2.210
Negativity/Pessimism	308	0.841	0.046
Entitlement/Insufficient Self-Control	308	0.208	− 0.459
Self-Sacrifice	308	0.307	− 0.469
Punitiveness	308	0.144	− 0.372
Unrelenting Standards	308	0.201	− 0.872
Approval-Seeking	308	− 0.080	− 0.579

## Results

In this part of the study, findings related to Personal Information Form, Eating Attitude Test, TEMPS-A and Young Schema Questionnaire are given.

Results regarding the scores obtained from the 14 schemas according to the gender variable are shown in Table 2. Women scored higher than men when the mean scores obtained from the Enmeshment/Dependence, Abandonment, Vulnerability to Harm or Illness, Failure and Negativity/Pessimism and Self-Sacrifice schemas are compared.

**Table 2:** Comparison of participants' scores received from the 14 schemas by gender variable

	n	M	SD	t	*p*
Emotional Deprivation					
Female	206	9.25	5.03	1.04	0.301
Male	102	8.66	4.10		
Social Isolation/Mistrust					
Female	206	15.81	6.78	1.02	0.307
Male	102	14.97	6.65		
Defectiveness/Shame					
Female	206	9.91	4.52	1.81	0.071
Male	102	8.97	3.79		
Emotional Inhibition					
Female	206	11.96	5.44	0.96	0.339
Male	102	11.33	5.25		
Enmeshment/Dependence					
Female	206	16.64	7.05	2.71	0.007*
Male	102	14.44	5.91		
Abandonment					
Female	206	9.61	4.64	4.00	< 0.001*
Male	102	7.57	3.14		
Vulnerability to Harm or Illness					
Female	206	12.59	5.83	3.47	0.001*
Male	102	10.29	4.64		
Failure					
Female	206	11.85	5.14	2.68	0.008*
Male	102	10.25	4.55		
Negativity/Pessimism					
Female	206	12.55	5.91	3.10	0.002*
Male	102	10.38	5.49		
Entitlement/Insufficient Self-Control					
Female	206	23.51	6.91	0.67	0.502
Male	102	22.90	8.52		
Self-Sacrifice					
Female	206	16.04	5.68	2.35	0.020*
Male	102	14.49	5.03		
Punitiveness					
Female	206	19.81	5.77	− 0.03	0.974
Male	102	19.83	5.36		
Unrelenting Standard					
Female	206	9.26	3.96	0.43	0.671
Male	102	9.06	3.92		
Approval-Seeking					
Female	206	18.93	6.25	1.04	0.297
Male	102	18.16	5.89		

*p < 0.05 Test Used: Independent Samples T-Test

Results regarding the scores obtained from the Eating Attitude Test and TEMPS-A according to the gender variable are shown in Table 3. Women scored higher than men when the mean scores obtained from the Eating Attitude Test (t(306) = 3.62, p < 0.05), Depressive Temperament subscale (t(306) = 2.61, p < 0.05), Cyclothymic Temperament subscale (t(306) = 3.79, p < 0.05) and Anxious Temperament subscale (t(306) = 4.12, p < 0.05) are compared.

**Table 3:** Comparison of participants' scores received from the eating attitudes and TEMPS-A by gender variable

	n	M	SD	t	*p*
Eating Attitude Test					
Female	206	23.13	14.51	3.62	< 0.001*
Male	102	17.37	9.78		
Depressive Temperament					
Female	206	6.76	4.00	2.61	0.010*
Male	102	5.48	4.13		
Cyclothymic Temperament					
Female	206	9.31	4.99	3.79	< 0.001*
Male	102	6.98	5.27		
Hyperthymic Temperament					
Female	206	10.86	4.87	0.34	0.737
Male	102	10.67	4.82		
Irritable Temperament					
Female	206	5.47	4.79	0.89	0.376
Male	102	4.94	5.08		
Anxious Temperament					
Female	206	10.17	6.63	4.12	< 0.001*
Male	102	6.78	7.10		

*p < 0.05 Test Used: Independent Samples T-Test

Results regarding the comparison of the relationship between the Eating Attitude Test and Young Schema Questionnaire are shown in Table 4.

**Table 4:** Comparison of the relationship between the Eating Attitude Test and the Young Schema Questionnaire

	Eating Attitude Test
Emotional Deprivation	0.246**
Social Isolation/Mistrust	0.208**
Defectiveness/Shame	0.408**
Emotional Inhibition	0.310**
Enmeshment/Dependence	0.388**
Abandonment	0.363**
Vulnerability to Harm or Illness	0.316**
Failure	0.302**
Negativity/Pessimism	0.327**
Entitlement/Insufficient Self-Control	0.098
Self- Sacrifice	0.174**
Punitiveness	0.214**
Unrelenting Standards	0.130*
Approval-Seeking	0.003

**p < 0.01, *p < 0.05 Test used: Pearson Correlation Test

Results regarding the comparison of the relationship between the Eating Attitude Test and TEMPS-A are shown in Table 5.

**Table 5:** Comparison of the relationship between the Eating Attitude Test and the TEMPS-A

	Eating Attitude Test
Depressive Temperament	0.166**
Cyclothymic Temperament	0.235**
Hyperthymic Temperament	0.081
Irritable Temperament	0.152**
Anxious Temperament	0.391**

**p < 0.01, *p < 0.05 Test used: Pearson Correlation Test

Results regarding the comparison of the relationship between the Eating Attitude Test and TEMPS-A are shown in Table 6.

**Table 6:** Comparison of the relationship between the Young Schema Questionnaire and the TEMPS-A

	Depressive Temperament	Cyclothymic Temperament	Hyperthymic Temperament	Irritable Temperament	Anxious Temperament
Emotional Deprivation	0.214**	0.268**	0.075	0.214**	0.268**
Social Isolation /Mistrust	0.207**	0.326**	0.123*	0.247**	0.133*
Defectiveness	0.268**	0.283**	00.004	0.240**	0.379**
Emotional Inhibition	0.166**	0.257**	0.137*	0.213**	0.265**
Enmeshment /Dependence	0.268**	0.305**	00.040	0.210**	0.374**
Abandonment	0.246**	0.301**	00.093	0.260**	0.351**
Vulnerability to Harm or Illness	0.262**	0.389**	00.087	0.270**	0.323**
Failure	0.201**	0.267**	− 0.083	0.144*	0.281**
Negativity/ Pessimism	0.320**	0.457**	00.044	0.274**	0.405**
Entitlement/ Insufficient Self Control	00.009	0.238**	0.339**	0.153**	− 0.024
Self- Sacrifice	0.219**	0.324**	0.197**	0.168**	0.157**
Punitiveness	0.138*	0.323**	0.176**	0.137*	0.093
Unrelenting Standards	0.079	0.186**	0.368**	0.205**	0.109
Approval-Seeking	0.072	0.176**	0.152**	0.116*	− 0.050

**p < 0.01, *p < 0.05 Test used: Pearson Correlation Test

Results regarding the prediction of eating attitude by schemas are shown in Table 7. Regression analysis was used Stepwise Method. In this method, variables that did not have significant predictor were excluded from the regression model. Defectiveness and Abandonment independent variables predicted eating attitude dependent variable (R^2^ = 0.19, *p* < 0.05). The independent variables in the model explained 19% of the total variance in the dependent variable of eating attitude. The relative order of influence according to beta is Defectiveness (β), Abandonment (β). The effect of Defectiveness and Abandonment subscales was positive. The variable that best explained the eating attitude was Defectiveness.

**Table 7:** Findings on the prediction of eating attitude by schemas

	B	SE	β	t	*p*	95%CI
(Constant)	6.96	1.81		3.83	< 0.001*	[3.39, 10.53]
Defectiveness	0.93	0.19	0.30	4.77	< 0.001*	[0.55, 1.31]
Abandonment	0.60	0.19	0.19	3.07	0.002*	[0.22, 0.98]
R = 0.44, R^2^ = .19 F = 36.18, *p* < 0.001						

*p < 0.05 Test used: Multiple Linear Regression Analysis

Results regarding the prediction of eating attitude by temperament are shown in Table 8. Regression analysis was used Stepwise Method. In this method, variables that did not have significant predictor were excluded from the regression model. Anxious temperament independent variable predicted eating attitude dependent variable (R^2^ = 0.15, *p* < 0.05). The independent variable in the model explained 15% of the total variance in the dependent variable of eating attitude. The effect of Anxious temperament was positive.

**Table 8:** Findings on the Prediction of Eating Attitude by Temperament

	B	SE	β	t	*p*	95%CI
(Constant)	14.42	1.15		12.49	< 0.001*	[12.15, 16.69]
Anxious Temperament	0.75	0.10	0.39	7.43	< 0.001*	[0.55, 0.95]
R = 0.39, R^2^ = .15 F = 55.22, *p* < 0.001						

*p < 0.05 Test used: Multiple Linear Regression Analysis

## Discussion

According to the results of the study, it was observed that women got higher scores from the Eating Attitude Test than men. Previous studies on eating disorders with a sample group consisting of only women supports these data [37–41]. In a study conducted with university students, it was shown that female students scored higher on the Eating Attitude Test average than male students [42]. A study conducted with mannequins stated that female models exhibited lower self-confidence, body satisfaction and significantly more eating disorder behaviors than men [43]. These studies examined in the literature support the results.

With the data of the study, it was concluded that there is a significant difference in cyclothymic, depressive and anxious temperament types according to the gender variable, and it is seen more in women than in men. In a study, men scored significantly higher in the hyperthymic temperament type, and women scored significantly higher in the irritable and anxious temperament type [31]. In another study by Vázquez et al. [44] in the population of Argentina, Korea, Hungary, Lebanon, Germany, Portugal and Spain, the scores obtained from the irritable and hyperthymic temperament types were higher in men, while the scores from the anxious, depressive and cyclothymic temperament types were higher in women. The studies support the results of the present study.

As a result of the study, it was observed that women scored higher than men on abandonment, vulnerability to harm or illness, failure, enmeshment/dependence, pessimism, and self-sacrifice. As a result of another study, it was stated that women got higher scores than men in 14 of 18 schemas [45]. Within the mentioned 14 schemas, there are schemas of abandonment, enmeshment/dependence, pessimism, failure, and self-sacrifice. The study argues that this difference in the schemas according to the gender variable may be caused by traumatic childhood experiences, etiological differences and gender roles [45].

When the relationship between early maladaptive schemas and eating attitudes was examined in the study, the following conclusions were reached: There is a moderate positive relationship between schemas of emotional inhibition, defectiveness, abandonment, enmeshment/dependence, vulnerability to harm or illness, failure, pessimism and eating attitude. There is a weak positive relationship between emotional deprivation, self-sacrifice, punitiveness, social isolation/mistrust and unrelenting standards schemas and eating attitudes. At the same time, defectiveness and abandonment schemas predict deterioration in eating attitudes, and it is seen that the scores obtained from the variable defectiveness schema explain this the most. In one study, it was stated that the defectiveness schema plays an important role in vomiting behavior in people with binge eating disorder. It has also been observed that people diagnosed with bulimia nervosa find themselves defective [38]. A study examining how the father-daughter relationship is in people diagnosed with eating disorders showed that abandonment and defectiveness schemes are at the forefront in these people [46]. A study examining the relationship between eating attitude and early maladaptive schemas found high scores on eating attitudes in young girls aged 17–18 on the Young Schema Scale were related to enmeshment, emotional deprivation, unrelenting standards, abandonment, vulnerability to harm or illness schemas [47]. In a study conducted with university and high school students, it was observed that people who display unhealthy behaviors in eating attitudes differ in areas of mistrust, vulnerability to harm or illness, insufficient self-control and unrelenting standards schema compared to those who display healthy behaviors [26]. In a study conducted with female university students in Turkey, it was found that emotional deprivation, self-sacrifice, and unrelenting standards schemas predicted difficulties in emotion regulation and eating attitude [48]. In another study conducted in Turkey, a direct effect was found between entitlement/insufficient self-control and punitiveness schemes and eating attitudes. At the same time, the others-directedness schema domain has a direct effect on eating attitudes. The reason for this is that the person directs his eating attitude according to social demands. For example; since the person seems more attractive to others and needs the approval of others, deterioration in eating attitudes may begin [49]. When we examine the findings of these studies conducted in the literature and summarized above, it is seen that the studies done overlap with the findings of the current study.

Studies conducted with people diagnosed with eating disorders [21], which result from a serious deterioration in their attitudes towards eating behavior, support the results of the current study and show the relationship between schemas and eating attitudes. As a result of a study conducted with people with and without an eating disorder diagnosis, people with bulimia nervosa, anorexia nervosa, and binge eating disorder got more schema scores in the areas of Inhibition, Overvigilance, Disconnection and Rejection, Impaired Limits and Impaired Autonomy and Performance compared to those without an eating disorder diagnosis. The study also revealed that people with binge eating disorder scored higher on Failure, Enmeshment/Dependence, and Entitlement/Grandiosity schemas than those diagnosed with bulimia nervosa [50]. As a result of the study of Unoka [51], who stated that there is a bidirectional relationship between early maladaptive schemas and eating disorders, it was seen that abandonment, Enmeshment / Dependence, emotional deprivation, emotional inhibition, and approval-seeking schemas were associated with eating disorders. Unoka et al. [52] stated that schemas play an important role in the development and maintenance of eating disorders, as a result of their study examining the effects of schemas on body mass index and eating disorders. As a result of this study conducted with individuals diagnosed with the restricting type and the binge-eating/purging type anorexia nervosa and bulimia nervosa, both types of anorexia nervosa have higher scores on Punitiveness, Self-Sacrifice and Unrelenting Standards schemas than bulimia nervosa. In addition, in binge-eating/purging type anorexia nervosa, the scores obtained from the entitlement and insufficient self- control schemas are higher than the restricting type. As a result of a study comparing the schemas in women who were diagnosed with bulimia nervosa and those who did not; It was observed that women diagnosed with bulimia nervosa had higher scores on insufficient self-control, failure and defectiveness schemes compared to others. Another important point of the study is that the early maladaptive schemas change according to the symptoms of these women diagnosed with bulimia nervosa. While the points taken from the emotion inhibition schema are high in the women who show binge eating behavior, the defectiveness schema plays an important role in the people who show vomiting behavior. Normal-weight bulimia nervosa patients define themselves as uncontrolled and flawed compared to others, but also see themselves as successful; Those who binge eat excessively define themselves as unsuccessful, but they state that they see themselves as less defective and uncontrolled than others [38]. Considering the studies mentioned above, it is seen that early maladaptive schemas are not only related to eating attitudes, but also differ between types of eating disorders.

As a result of the present study, it was seen that there was a moderate positive relationship between the deterioration of people's eating attitudes and anxious temperament type, and a weak positive relationship with cyclothymic and irritable temperament. At the same time, the results show that anxious temperament positively predicts deterioration in eating attitudes. Ramacciotti et al. [53], it was observed that people with binge eating and vomiting behaviors showed more cyclothymic temperament characteristics than the healthy group, however, it was observed that they showed hyperthymic and depressive temperament features prominently. In another study, a significant relationship was found between cyclothymic temperament and restrictive food intake disorder. Cyclothymic temperament was observed in 25.2% of students with eating disorders, and it was stated that the risk of developing eating disorders in female students with cyclothymic temperament could be 3.75 times higher than the others [54]. Another study by Chapuis-de-Andrade et al. [55] stated that there are deteriorations in eating behavior, especially in women with cyclothymic temperament. The study showed that eating-compensatory behaviors were associated with anxious and irritable temperaments at a low level and eating-compensatory behaviors were associated with affective temperament types.

As a result of the present study, when the relationship between early maladaptive schemas and temperament types is examined, the results are as follows; there is a weak positive relationship between the depressive temperament type and the schemas of emotional deprivation, social isolation/mistrust, defectiveness, emotional inhibition, enmeshment/dependence, abandonment, vulnerability to harm or illness, failure, self-sacrifice and punitiveness, and a moderate positive relationship with the pessimism schema. It has been observed that there is a relationship between cyclothymic temperament type and all schema domains. While there is a moderate positive relationship with social isolation/mistrust, enmeshment/dependence, abandonment, vulnerability to harm or illness, pessimism, self-sacrifice, punitiveness and unrelenting standards, there is a weak positive relationship with other schemas. It was observed that irritable temperament type had a weak positive relationship with all schemas. There was a moderate positive relationship between hyperthymic temperament type and unrelenting standards and entitlement/insufficient self-control schemas, while a weak positive relationship was observed between social isolation/mistrust, emotional inhibition, self-sacrifice, punitiveness and approval seeking schemas. Finally, anxious temperament type was found to be associated with low levels of emotional deprivation, social isolation, emotional inhibition, failure, and self-sacrifice; it was found to be moderately and positively associated with schemas of defectiveness, dependence, abandonment, vulnerability to harm or illness, and pessimism. There are few studies in the literature examining the relationship between early maladaptive schemas and affective temperament. The studies found mostly dealt with this relationship with bipolar disorder and borderline personality disorder. Nabizadeh-Chianeh et al. [56] who worked with 3 groups diagnosed with healthy individuals, borderline personality disorder and bipolar stated that there is a significant relationship between affective temperaments and early maladaptive schemas in borderline personality disorder. The study revealed that people diagnosed with borderline personality disorder scored higher in all temperament types and early maladaptive schemas compared to bipolar and healthy individuals. Compared to healthy individual, individuals diagnosed with bipolar have higher scores on abandonment, social isolation/mistrust, vulnerability to harm or illness, enmeshment, entitlement/insufficient self-control, approval seeking, and punitiveness schemes compared to healthy individuals [56]. Another study trying to understand the similarities and differences between bipolar disorder and borderline personality disorder stated that borderline individual scored higher on schemas and temperament than bipolar and healthy individual. Bipolar patients, on the other hand, scored significantly higher on cyclotomic temperament and insufficient self-control schema [57]. These studies show that there is a relationship between early maladaptive schemas and temperament, and that this relationship may play an important role in mental illnesses that individuals may develop.

## Conclusion

In the future, it is thought that to explain the relationship between temperament and schemas and eating disorders, only working with people diagnosed with eating disorder, and the results will strengthen the studies in the literature and the results of these studies. At the same time, it is thought that the use of this model in studies on eating attitudes and eating disorders will contribute to the literature due to the limited number of studies with the Akiskal’s affective temperament model. Lastly, when the literature is examined, it is seen that there are many studies that examine the relationship between temperament and schemas and Axis 1 and 2 disorders separately. At the same time, although there are studies examining the relationship between these two concepts on pathology, their number is quite low. While examining the formation and maintenance of eating attitudes and other disorders, the relationship between temperament and schemas will play an important role for us as well as the relationship between them. For this reason, it is thought that it will be useful to evaluate these two concepts together in future studies.

## Data Availability

The datasets used and/or analyzed during the current study are available from the corresponding author on reasonable request.

## Abbreviations

YSQ-SF3: Young Schema Questionnaire—Short Form 3
TEMPS-A: Temperament Evaluation of Memphis, Pisa, Paris and San-Diego Autoquestionnaire
